# Identification of a Twelve Epithelial-Mesenchymal Transition-Related lncRNA Prognostic Signature in Kidney Clear Cell Carcinoma

**DOI:** 10.1155/2022/8131007

**Published:** 2022-03-23

**Authors:** Qi-Dong Xia, Yuan Zhang, Li-Sha Li, Jun-Lin Lu, Yang Xun, Jian-Xuan Sun, Jin-Zhou Xu, Chen-Qian Liu, Yu-Chao Lu, Deng He, Shao-Gang Wang

**Affiliations:** ^1^Department and Institute of Urology, Tongji Hospital, Tongji Medical College, Huazhong University of Science and Technology, No. 1095 Jiefang Avenue, 430030 Wuhan, China; ^2^Department of Gynecology and Obstetrics, Tongji Hospital, Tongji Medical College, Huazhong University of Science and Technology, No. 1095 Jiefang Avenue, 430030 Wuhan, China

## Abstract

**Background:**

Epithelial-mesenchymal transition (EMT) plays a vital role in tumor metastasis and drug resistance. It has been reported that EMT is regulated by several long noncoding RNAs (lncRNAs). We aimed to identify EMT-related lncRNAs and develop an EMT-related lncRNA prognostic signature in kidney renal clear cell carcinoma (KIRC).

**Materials and Methods:**

In total, 530 ccRCC patients with 611 transcriptome profiles were included in this study. We first identified differentially expressed EMT-related lncRNAs. Then, all the samples with transcriptional data and clinical survival information were randomly split into training/test sets at a ratio of 1 : 1. Accordingly, we further developed a twelve differentially expressed EMT-related lncRNA prognostic signature in the training set. Following this, risk analysis, survival analysis, subgroup analysis, and the construction of the ROC curves were applied to verify the efficacy of the signature in the training set, test set, and all patients. Besides, we further investigated the differential immune infiltration, immune checkpoint expression, and immune-related functions between high-risk patients. Finally, we explored the different drug responses to targeted therapy (sunitinib and sorafenib) and immunotherapy (anti-PD1 and anti-CTLA4).

**Results:**

A twelve differentially expressed EMT-related lncRNA prognostic signature performed superior in predicting the overall survival of KIRC patients. High-risk patients were observed with a significantly higher immune checkpoint expression and showed better responses to the targeted therapy and immunotherapy.

**Conclusions:**

Our study demonstrates that the twelve differentially expressed EMT-related lncRNA prognostic signature could act as an efficient prognostic indicator for KIRC, which also contributes to the decision-making of the further treatment.

## 1. Introduction

As a fundamental and reversible biological process in physiological and pathological conditions, epithelial-mesenchymal transition (EMT) has been identified in three different types [1]. Type I is majorly involved in growth and development, especially in embryogenesis and organ development [2]. Type II is majorly involved in wound healing [2], and type III is regarded as one of the essential processes in tumorigenesis, progression, tumor metastasis, and drug resistance [3, 4]. The changing phenotype from epithelial cells to mesenchymal cells obtains great migratory and even invasion ability. Researches to date have reported that EMT plays an essential role in invasion-metastasis cascade in tumor migration, contributing to the diffusion of cancer cells to the surrounding stromal environment and followed intravasation that also means hematogenous metastasis and finally resulted in the metastasis to the distant organ [5, 6].

Furthermore, the EMT mechanism is also involved in mediating drug resistance [7–9]. For example, though tyrosine kinase inhibitors (TKIs) have been extensively applied in targeted therapy in several tumor types, the most significant limitation lies in cancer resistance. Notably, it has been reported that EMT induction through YAP/FOXM1 axis contributes to EGFR inhibitor resistance [10]. Besides, nerve growth factor (NGF) can stimulate the EMT mechanism in triggering erlotinib resistance [11]. Hence, the significant role of EMT in cancer drives researchers to explore the EMT-related genes, especially EMT regulator genes [12, 13]. Meanwhile, Zhao et al. collected all EMT genes with experimental verification and set up an EMT gene database named dbEMT [14, 15]. However, it is essential to continue exploring the EMT-related genes, which may be helpful to discover novel therapeutical targets or relieve the current crisis of drug resistance.

Kidney cancer is the 6th most common cancer in both sexes and the most common urogenital tumor [16], accounting for approximately 2-3% of all malignancies and 90% of all diagnosed renal parenchymal malignancies [16, 17], claiming 14,830 lives with 73,750 new confirmed cases in the USA in 2020 [16]. Kidney renal clear cell carcinoma (KIRC) is the predominant pathological subtype of all kidney cancer, accounting for approximately 85% of renal cancer [18, 19], also considered one of the most invasive diseases associated with a high mortality rate with the form of metastasis [20]. As KIRC is not sensitive to radiation, hormone, or cytotoxic therapy, tyrosine kinase inhibitors (TKIs) such as sunitinib and sorafenib targeting vascular endothelial growth factor (VEGF) pathway play an essential role in the current clinical treatment as the first-line targeted therapy [21, 22]. In recent years, immune checkpoint inhibitors (ICIS) that block PD-1/PD-L1 or CTLA-4/T-cell suppressor inhibitory have also shown excellent performance in the therapy of KIRC [23], especially with the combination of VEGF-directed therapy [24]. Also, immunotherapy-combined therapy has replaced TKI's first-line targeted therapy as a first-line treatment in the latest European Association of Urology (EAU) guidelines for clear cell metastatic renal cell carcinoma (cc-mRCC) [25]. The overall survival (OS) rate for localized KIRC reaches 95%, but those KIRC patients with metastasis and drug resistance had a poor OS [26]. Thus, it is essential to discover precise prognostic biomarkers associated with drug resistance in KIRC.

The long noncoding RNA (lncRNA), defined as RNA transcripts with longer than 200 nucleotides and little protein-coding ability [27], have been found with robust prognostic value in KIRC [28]. Interestingly, lncRNAs also play a significant role in the regulation of EMT [29, 30]. Combined with the above, EMT-derived cancer metastasis and drug resistance resulted in poor prognosis of patients, and KIRC is a typical tumor type that its metastasis is usually challenging to detect and its resistance to TKIs is a thorny problem clinical treatment. Both these resulted in a poor prognosis. Thus, lncRNAs may play a critical role as a regulator factor in EMT in KIRC. In our study, we first identified differentially expressed EMT lncRNAs in KIRC, then constructed and verified the EMT-related lncRNA signature, and further explored and found that the prognostic signature was significantly associated with immune infiltration, immune functions, immune checkpoints, and, more importantly, drug response to targeted therapy and immunotherapy.

## 2. Materials and Methods

### 2.1. Data Sources

The transcriptional expression profiles and corresponding clinical information contained survival time, and clinicopathological characteristics for the KIRC were retrieved from TCGA-GDC (https://portal.gdc.cancer.gov/). Notably, the transcriptional profiles were downloaded in the fragments per kilobase of per million (FPKM) format and then merged as an eligible matrix. The corresponding clinical data was downloaded in the bcr xml format and then merged as a clinical information matrix. Besides, the gene annotation files were acquired from the Ensembl database (http://asia.ensembl.org/index.html) to transfer the Ensembl ID in the matrix to the gene symbol and annotate genes for its transcripts type, such as mRNA and lncRNA. More importantly, EMT-related gene list was downloaded from the dbEMT 2.0 database (http://dbemt.bioinfo-minzhao.org/). All the downloaded data was proceeded by R program version 4.1.0 [31].

### 2.2. Differentially Expressed EMT-Related mRNAs and EMT-Related lncRNAs

According to the tissue source, we sorted the transcriptional data: normal adjacent tumor tissue as “normal” and tumor tissue as “tumor.” Then, we divided the transcriptional atlas of KIRC patients into two matrices: one for mRNA expression atlas and another for lncRNA expression atlas. Following this, we exported the expression atlas of EMT-related mRNAs according to the gene list downloaded from dbEMT 2.0. We performed the Pearson correlation test between expression of EMT-related mRNAs and lncRNAs, set the ∣correlation coefficient | >0.5, and adjusted *p* value < 0.001 as the filter to identify EMT-related lncRNAs. Subsequently, we applied the Wilcoxon test between normal tissue and tumor tissue to discover significantly differentially expressed EMT-related mRNAs and EMT-related lncRNAs. GO enrichment analysis and KEGG enrichment analysis were employed to check the potential functions and pathways influenced by the differentially expressed EMT-related mRNAs.

### 2.3. Random Grouping and Signature Construction

Having identified and screened differentially expressed EMT-related lncRNAs, we merged it with its corresponding clinical survival data. All the samples with transcriptional data and clinical survival information were randomly split into training/test sets at a ratio of 1 : 1. Following this, we performed univariate Cox regression in the training group to identify prognostic EMT-related lncRNAs with a significance of *p* < 0.05. Subsequently, we applied LASSO regression to avoid overfitting and screen appropriate variables. Finally, a survival-predicting signature was constructed by the multivariate Cox proportional hazards model. Notably, a risk score formula was accordingly created based on the signature:

(1) $$\begin{array}{c} \text{Risk score}=\overset{N}{\underset{i=1}{\sum }}\left(\text{Exp}\left(i\right)∙\text{coe}\left(i\right)\right), \end{array}$$

where *N* is the number of differentially expressed EMT-related lncRNAs in the multivariate Cox proportional hazards model; Exp(*i*) is the expression value normalized by FPKM of the *i*th lncRNA in the signature, and coe(*i*) is the estimated regression coefficient of it. Besides, all samples in both the training set and test set obtained a risk score calculated by the predict function in the R program, and we set the medium value of the risk score in the training set as the cut-off value to stratify patients with KIRC that the higher risk score represented a high risk, and the lower risk score was grouped into low risk.

### 2.4. Signature Validation

We first described the distribution of the risk scores and the proportion of risk stratification in the training set, the test set, and all samples and visualized the survival status of each patient with different risk scores through risk analysis, and then, we performed the Kaplan-Meier method to plot the survival curves for the patients in the training set, test set, and all patients. The log-rank test was carried out to examine the survival-predicting availability of this signature at the same time. Following this, we sort the clinicopathologic information of each patient, considering age, gender, stage, grade, and risk score as the alternative prognostic factors, and then compared the predicting capability of these prognostic factors by the 5-year multivariate ROC curves; simultaneously, the area under the curve (AUC) for each prognostic factors including age, gender, stage, grade, and risk score was calculated and compared with each other in the training set, test set, and all patients.

### 2.5. EMT-Related mRNA-EMT-Related lncRNA Interaction Network

We were interested in the correlation between these EMT-related lncRNAs included in the signature and their correlated EMT-related mRNAs. Thus, we extracted the correlation between these two from the results of the Pearson correlation test and visualized it as an interaction network by using Cytoscape (version 3.8.0) [32].

### 2.6. Clinical Correlation and Subgroup Analysis

Further investigating the differences of these clinicopathologic characteristics between high- and low-risk groups might contribute to learning the potential correlation between the risk score and clinical characteristics. All patients were divided into two groups as a high- or low-risk group; and then, the *χ*2 test was applied to examine the difference of clinicopathologic status between these two groups. Moreover, all patients were divided into subgroups according to each clinicopathologic characteristic, including age (age > 65 and ≤ 65), gender (male and female), stage (stage I-II and stage III-IV), and grade (G1-2 and G3-4). Then, survival analysis was employed to test the efficacy of this signature in all different subgroups.

### 2.7. Immune Infiltration, Immune Functions, Immune Checkpoint Expression, and Drug Response

Having verified the efficacy of this signature, we were interested in the immune infiltration, immune-related functions, and immune checkpoint expression between high- and low-risk patients. Thus, we separately applied the Wilcoxon test to compare the immune infiltration and immune checkpoint expression status. Immune-related functions were investigated by single set gene set enrichment analysis (ssGSEA) [33]. Also, though high-risk patients showed a poor prognosis, we wondered whether high-risk patients had other alternative therapeutic choices, such as targeted therapy and immunotherapy. Thus, the drug response to sunitinib and sorafenib for each patient was predicted by R package “Prophetic,” and drug response to immunotherapy was predicted by the submap algorithm [34]. Finally, each drug response was compared between high- and low-risk patients.

## 3. Results

### 3.1. Basic Characteristics

The study flow was shown in the graphic abstract. A total of 611 transcriptome profiles containing 72 normal tissues and 539 tumor tissues from 530 KIRC patients were downloaded and sorted. For those samples sequenced multiple times, we took the average of them as their transcriptional data. Besides, all samples were randomly split into training and test set at a ratio as 1 : 1, and the characteristics of the samples in the training set, test set, and all samples are shown as Table 1; the *χ*^2^ test or Fisher's exact test was applied to explore the heterogeneity between the training set and test set. It seemed that there was no significant difference between the training set and the test set.

### 3.2. Differentially Expressed EMT-Related mRNAs and EMT-Related lncRNAs

**Figure 1:**
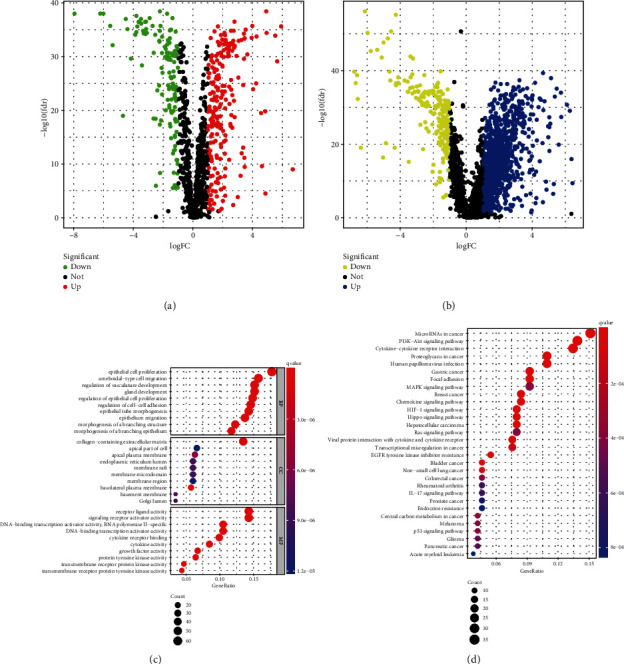
Differentially expressed EMT-related mRNAs and EMT-related lncRNAs. (a) Differentially expressed EMT-related mRNAs with ∣logFC | >1 and fdr < 0.05, (b) differentially expressed EMT-related lncRNAs with ∣logFC | >1 and fdr < 0.05, (c) GO enrichment analysis, and (d) KEGG enrichment analysis.

A total of 1184 EMT-related genes were downloaded from the dbEMT 2.0 database. Then, we extracted the transcriptional expression atlas of 1135 EMT-related mRNAs and identified 2380 EMT-related lncRNAs with ∣Cor | >0.5 and *p*.adj < 0.001. Following this, 358 differentially expressed EMT-related mRNAs (Figure 1(a)) and 1339 differentially expressed EMT-related lncRNAs (Figure 1(b)) with ∣logFC | >1 and FDR < 0.05 were screened by the Wilcoxon test for further analysis. GO enrichment analysis showed that the most significantly enriched function for these differentially expressed EMT-related mRNAs is the EMT (Figure 1(c)). KEGG analysis revealed that these EMT-related mRNAs play an important role in several tumor-related pathways (Figure 1(d)).

## 4. Signature Construction and Signature Verification

**Figure 2:**
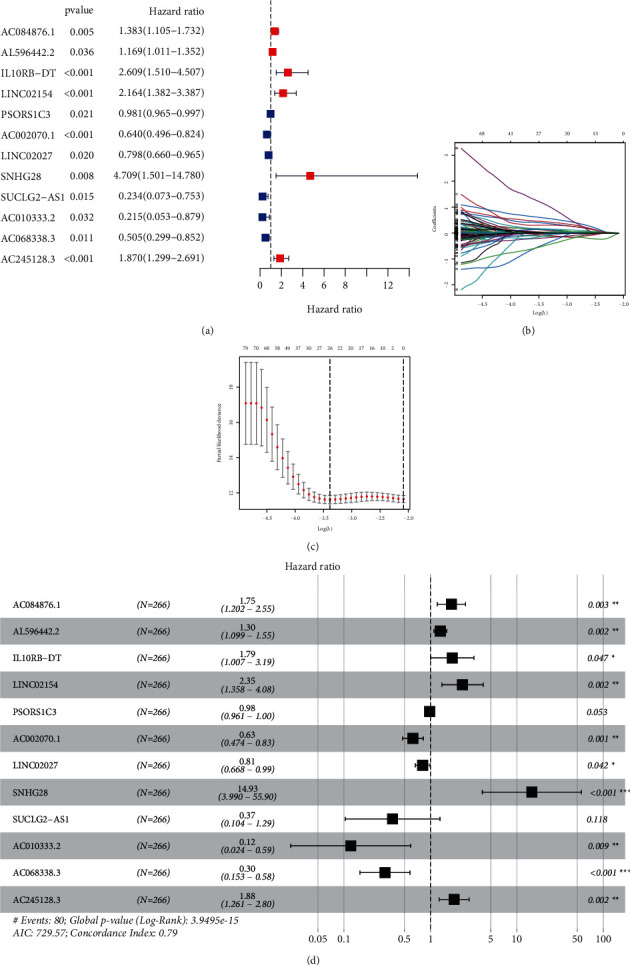
Development of a twelve differentially expressed EMT-related lncRNA signature. (a) Univariate cox regression of these twelve EMT-related lncRNAs, (b) variables going to zero as we increase the penalty (lambda) in the objective function of the LASSO, (c) 10-fold cross-validation for tuning parameter selection in the LASSO model, −3.5 < lambda.min < −3.0, and there were 26 variables (lncRNAs) left, and (d) the twelve differentially expressed EMT-related lncRNA prognostic signature.

**Figure 3:**
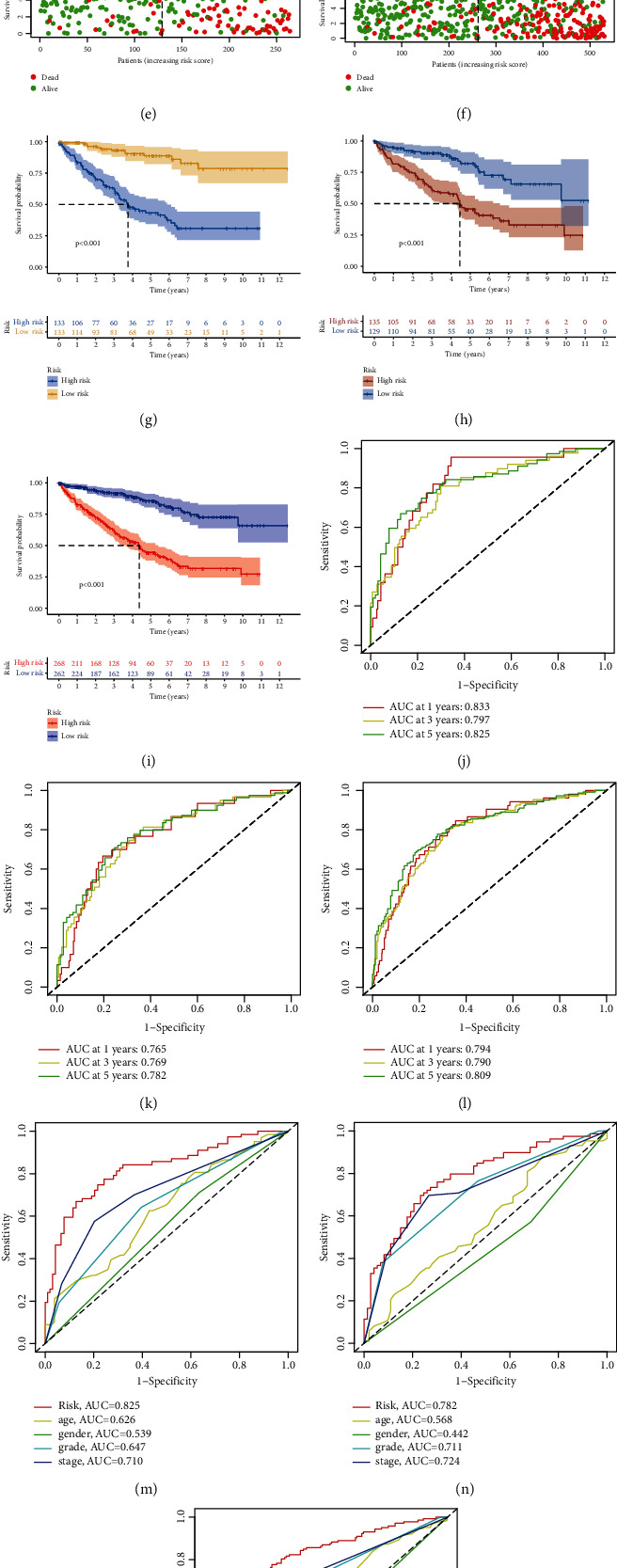
Primary validation of this prognostic signature. (a) Risk plot of training set, (b) risk plot of test set, (c) risk plot of all patients, (d) survival status in training set, (e) survival status in test set, (f) survival status in all patients, (g) survival curve of training set, (h) survival curve of test set, (i) survival curve of all patients, (j) 1-year, 3-year, and 5-year ROC curves in training set, (k) 1-year, 3-year, and 5-year ROC curves in test set, (l) 1-year, 3-year, and 5-year ROC curves in all patients, (m) 5-year multivariate ROC curves in training set, (n) 5-year multivariate ROC curves in test set, and (o) 5-year multivariate ROC curves in all patients.

In the training set, we discovered 265 prognostic differentially expressed EMT-related lncRNAs through univariate cox regression with a *p* value < 0.05. The univariate Cox regression of the lncRNAs in the final signature is shown in Figure 2(a). Then, the LASSO regression was carried out to avoid overfitting and screened 26 appropriate EMT-related lncRNAs to conduct further multivariate Cox proportional hazards model (Figures 2(b) and 2(c)). Finally, we developed a twelve-EMT-related lncRNA survival-predicting signature (Figure 2(d)), and their detailed information is shown in Table 2. The risk score calculating formula was established as mentioned in the Materials and Methods. Then, each patient acquired a risk score and corresponding risk stratification according to the medium value of risk score in the training set. The distribution status of the risk score in the training, test, and all patients is shown in Figures 3(a)–3(c). The survival status of each patient with different risk levels is shown in Figures 3(d)–3(f). Following this, the survival analysis was carried out as shown in Figures 3(g)–3(i). The 1-year, 3-year, and 5-year ROC curves of the risk score in the training set, the test set, and all patients are shown in Figures 3(j)–3(l). More importantly, the multivariate 5-year ROC curves in different sets were assembled to compare the efficacy of the risk score to other common-used clinicopathological characteristics, and the risk score showed the best performance with the most extensive area under curves (AUC) in the training set (Figure 3(m)), test set (Figure 3(n)), and all patients (Figure 3(o)).

## 5. Interaction Network and Clinical Correlation

**Figure 4:**
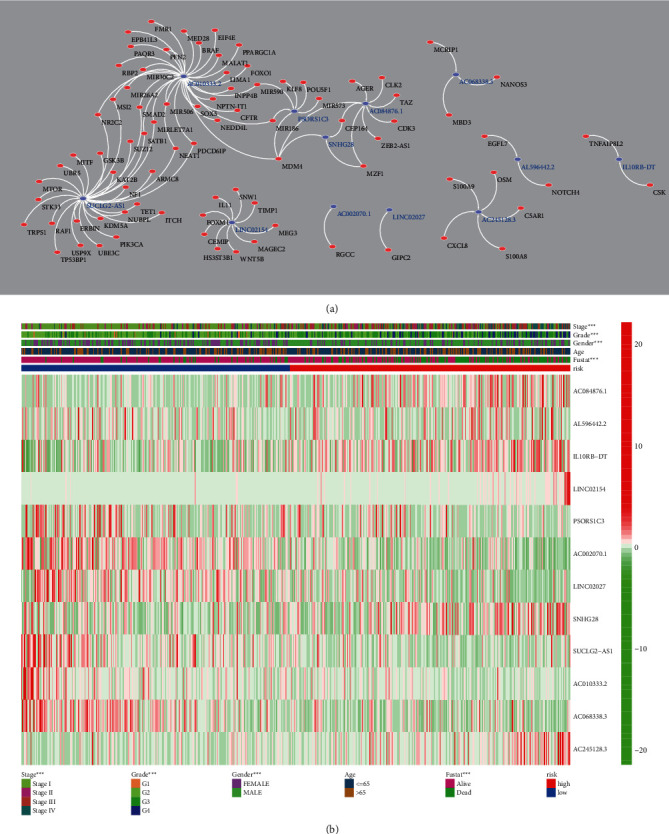
EMT gene-EMT-related lncRNAs interaction network and expression atlas of the twelve EMT-related lncRNAs. (a) EMT gene-EMT-related lncRNA interaction network and (b) expression status of the EMT-related lncRNAs between high/low risk and the clinical correlation of risk stratification.

Having constructed the prognostic signature, we established the EMT-related mRNA-EMT-related lncRNA interaction network according to the results of the initial Pearson correlation test. The network is shown in Figure 4(a). The blue nodes represented the twelve EMT-related lncRNAs, while the left red nodes represented EMT-related mRNAs coexpressed with these lncRNAs. The clinical correlation checked by the *χ*^2^ tests and the expression status of these twelve EMT-related lncRNAs were visualized in a heat map as shown in Figure 4(b). Notably, the risk level is associated with the distribution of gender, stage, grade, and survival status of these KIRC patients (Figure 4(b)).

### 5.1. Subgroup Analysis

**Figure 5:**
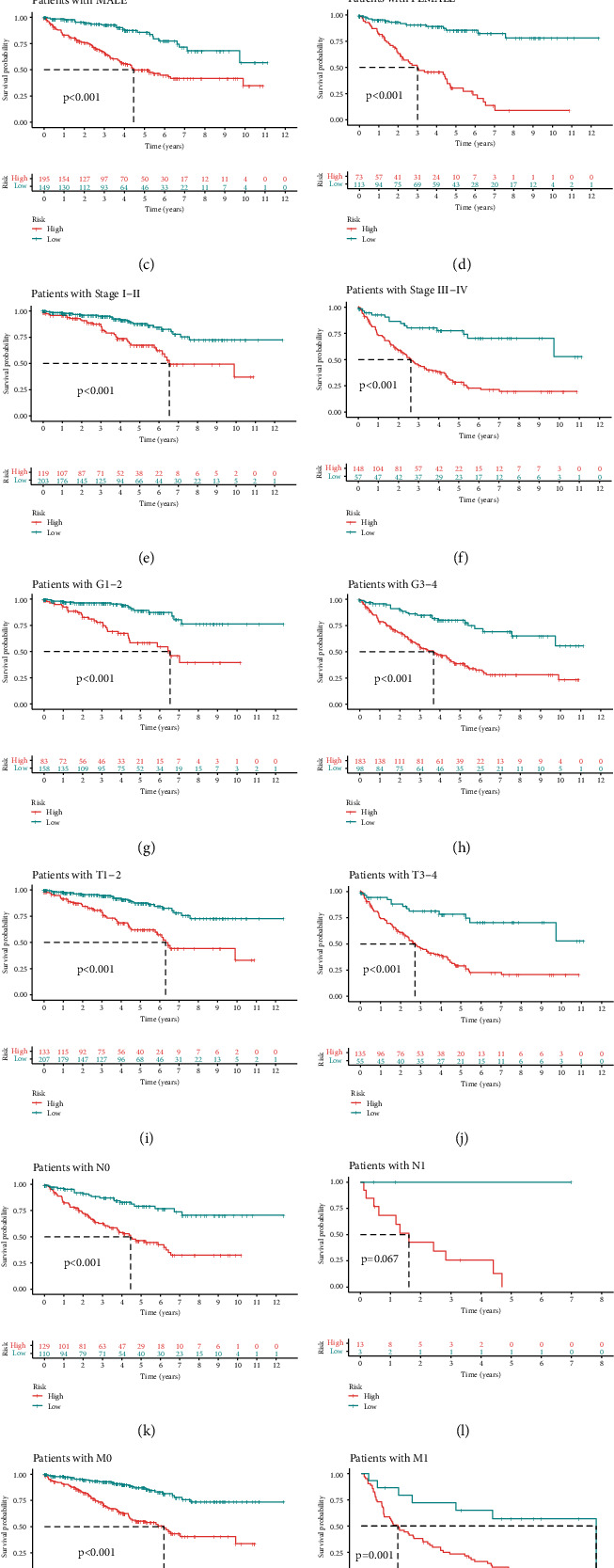
Subgroup analysis. (a) Survival differences distinguished by the risk score in patients with age > 65, (b) survival differences distinguished by the risk score in patients with age ≤ 65, (c) survival differences distinguished by the risk score in male patients, (d) survival differences distinguished by the risk score in female patients, (e) survival differences distinguished by the risk score in patients with stage I-II, (f) survival differences distinguished by the risk score in patients with stage III-IV, (g) survival differences distinguished by the risk score in patients with G1-2, (h) survival differences distinguished by the risk score in patients with G3-4, (i) survival differences distinguished by the risk score in patients with T1-2, (j) survival differences distinguished by the risk score in patients with T3-4, (k) survival differences distinguished by the risk score in patients with N0, (l) survival differences distinguished by the risk score in patients with N1, (m) survival differences distinguished by the risk score in patients with M0, and (n) survival differences distinguished by the risk score in patients with M1.

Because the risk stratification by this signature showed a significant correlation with gender, grade, and stage (Figure 4(b)), we divided all patients into several subgroups by their clinical characteristics to explore the universality of this signature, including age (Figures 5(a) and 5(b)), gender (Figures 5(c) and 5(d)), stage (Figures 5(e) and 5(f)), grade (Figures 5(g) and 5(h)), T stage (Figures 5(i) and 5(j)), N stage (Figures 5(k) and 5(l)), and M stage (Figures 5(m) and 5(n)). Interestingly, the risk stratification by this signature was generally verified effective in all subgroups that high-risk patients keep a significantly worse overall survival, suggesting the great universality of this prognostic signature.

### 5.2. Immune Infiltration, Immune Checkpoint Expression, Immune-Related Functions, and Drug Response

**Figure 6:**
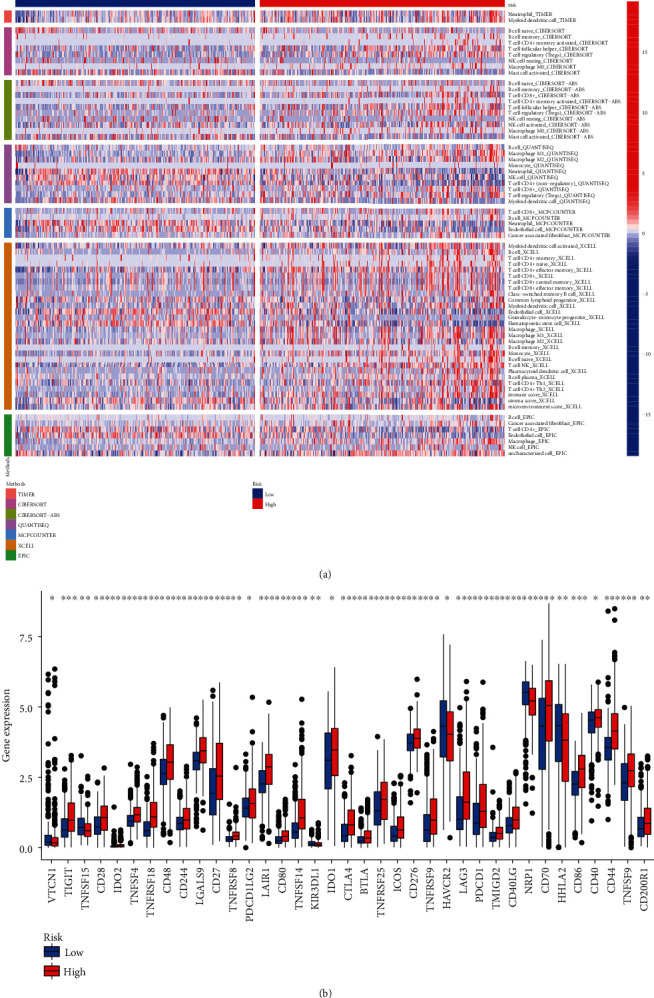
Immune infiltration and immune checkpoint expression. (a) Significant differential immune infiltration calculated by several acknowledged methods and (b) differential expression status of immune checkpoint between high- and low-risk patients.

**Figure 7:**
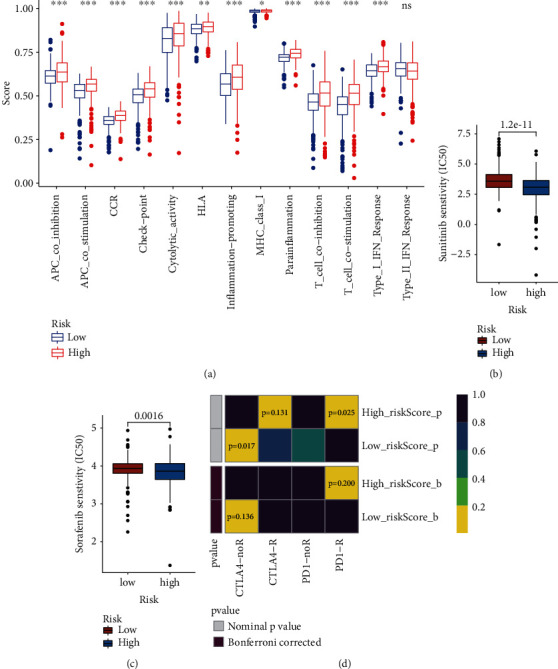
Immune-related functions and drug response. (a) Differential active immune-related functions between high-risk and low-risk patients, (b) high-risk patients show a better response to sunitinib, (c) high-risk patients show a better response to sorafenib, and (d) high-risk patients show a better response to both anti-PD1 and anti-CTLA4 immunotherapy.

Differential immune infiltration between high- and low-risk patients calculated by several different methods is shown in Figure 6(a), and the differential expression of immune checkpoint between high risk and low-risk patients is shown in Figure 6(b). Interestingly, almost all the immune checkpoints were a higher expression in high-risk patients, which might account for the poor OS for high-risk patients. The results of ssGSEA revealed differential activated immune-related functions between high- and low-risk patients (Figures 7(a)-7(d)). More importantly, the high-risk patients showed a more sensitive drug response to either sunitinib or sorafenib. Finally, considering the differential immune infiltration and immune-related functions, we explored and found high-risk patients more suitable for anti-PD1 immunotherapy and well responded to immunotherapy.

## 6. Discussion

It has been reported that the five-year cancer-specific survival (CSS) rate of ccRCC was 91%, 74%, 67%, and 32% separately for AJCC stages I, II, III, and IV, respectively [35]. The OS of differential individuals is heterogeneous, and it is challenging to predict the prognosis of patients with KIRC accurately using the AJCC stage, grade, and pathological TNM stage. With the rapid development of molecular sciences of ccRCC biology and high-throughput sequencing, numerous fresh biomarkers for predicting the prognosis or therapeutic targets have been designed and raised in recent years [28, 36]. lncRNAs, as a complement to genes or microRNAs, have been shown to regulate many cell functions, such as proliferation, apoptosis, invasion, and metastasis [37]. lncRNAs are involved in major oncogenic events in genitourinary malignancies, including invasiveness and recurrence in ccRCC [38]. Therefore, it is essential to establish a potential reliable lncRNA signature to predict the prognosis of ccRCC patients.

Epithelial-mesenchymal transition (EMT), first introduced by Newgreen et al. in 1979 [39], was discovered to play an essential role in tumor metastasis [3]. Notably, nearly 80% to 90% of the tumors come from epithelial cells, which are closely connected and cannot move, so it is challenging to metastasize. However, the activation of epithelial-mesenchymal transformation can provide tumor cells with the ability of migration, infiltration, and invasion [4]. Once they reach distant organs, these mesenchymal cells will return to the characteristics of epithelial cells through mesenchymal-epithelial transition (MET) and restore the ability of proliferation and form secondary foci in the distance [4]. Therefore, primary tumors need to conduct EMT, to obtain the ability to migrate before they can metastasize. Besides, the researchers also found that the invasion and metastasis of drug-resistant cancer cells were significantly enhanced, suggesting that EMT played an essential role in tumor drug resistance [40–42]. Ashrafizadeh et al. summarized the vital role of epithelial-mesenchymal cells in paclitaxel and docetaxel resistance [1].

Furthermore, researchers focused on the cell plasticity with the stemness of tumor cells [8]. They believed that once driven by EMT, tumor cells obtain more heterogeneity and plasticity. Therefore, conventional therapy (including radiotherapy, chemotherapy, and targeted therapy) is difficult to eradicate tumor cells that enter the cancer stem cells (CSC) state by activating EMT. Thus, it is quite challenging to prevent drug resistance and recurrence or progression after treatment.

In the present study, we focused on the EMT-related lncRNAs in KIRC and identified and developed a twelve-EMT-related lncRNA prognostic signature in KIRC. Following this, we verified the prognostic signature in the test set by log-rank test, risk analysis, subgroup analysis, and time-dependent multivariate ROC curves. Interesting, in the subgroup analysis, this prognostic signature showed an outstanding performance in almost all clinicopathological subgroups, which indicate the universality of our prognostic signature. Besides, in the comparison for the prognostic signature with common-used clinicopathological characteristics, including age, gender, grade, and stage, this prognostic signature showed a superior AUC. All these verified the accuracy and sensitivity of this EMT-related lncRNA signature. More importantly, though high-risk patients stratified by this signature had a worse OS, we found that the high-risk patients showed a more sensitive drug response to both sunitinib and sorafenib, which might contribute to the clinical decisions.

Furthermore, the differential immune infiltration, immune checkpoint expression, and immune-related functions were engaging, consistent with the current opinion. Zhou et al. reported that the activation of EMT was associated with the upregulation of the immune checkpoint expression (especially PD-L1) [7], which mean EMT induced immune escape to promote cancer progression. Also, Nilsson et al. summarized the association between EMT, MET, and the tumor microenvironments that the changes of tumor plasticity resulted in differential immune infiltrations and differential immune response [10]. These well accounted for the better response to the immunotherapy in high-risk patients than low-risk patients.

Besides, we constructed an EMT-related lncRNA-EMT gene network, which may provide potential regulatory mechanisms of EMT. Further experimental examination is needed to explore the interaction network in the future. Notably, in these twelve EMT-related lncRNAs, *AC084876.1* has been identified as glycolysis-related lncRNA with prognostic value in KIRC by Cao et al. [43]. *IL10RB-DT* has been reported associated with immune infiltration in KIRC [44]. *LINC02154* was discovered significantly differentially expressed between normal and tumor tissues in laryngeal cancer [45, 46]. *PSORS1C3* is a significant lncRNA in immune-mediated diseases that have been identified regulated by glucocorticoids (GC) and showed great prognostic values in bladder cancer [47, 48]. *SUCLG2-AS1* has been reported with prognostic value in clear cell carcinoma, gastric cancer, and triple-negative breast cancer [49–51]. *AC068338.3* has been reported as a significant prognostic factor associated with immune infiltration in lung adenocarcinoma [52, 53]. However, our knowledge about these lncRNAs is far from enough, and further investigation is needed in the future.

There are some limitations in our study. Firstly, there lacks an independent validation set. Besides, we revealed a twelve differentially expressed-EMT-related-lncRNA prognostic signature for ccRCC with sufficient bioinformatical analysis and statistical-method based verification, which lacks relevant experimental verification. Further experimental verification is required to explore the molecular mechanism behind the association between this signature with immune infiltration, immune checkpoint expression, drug response to targeted therapy and immunotherapy.

## 7. Conclusions

In summary, we successfully developed a twelve differentially expressed EMT-related lncRNA prognostic signature, which could robustly predict the overall survival and prognosis of KIRC patients. The prognostic signature performed best compared to other frequently used prognostic clinicopathologic factors in predicting the overall survival. However, large-scale, multicenter prospective researches need to be carried out to confirm our results in the future.

## Figures and Tables

**Table 1:** Clinicopathological characteristics of KIRC patients enrolled in this study.

*N*	Overall	Test	Train	*p*
	530	264	266	
Age (mean (SD))	60.56 (12.14)	60.62 (11.97)	60.51 (12.33)	0.914
Gender = female/male (%)	186/344 (35.1/64.9)	94/170 (35.6/64.4)	92/174 (34.6/65.4)	0.877
Grade (%)				0.063
G1	14 (2.6)	7 (2.7)	7 (2.6)	
G2	227 (42.8)	100 (37.9)	127 (47.7)	
G3	206 (38.9)	107 (40.5)	99 (37.2)	
G4	75 (14.2)	47 (17.8)	28 (10.5)	
GX	5 (0.9)	1 (0.4)	4 (1.5)	
Unknown	3 (0.6)	2 (0.8)	1 (0.4)	
Stage (%)				0.121
Stage I	265 (50.0)	126 (47.7)	139 (52.3)	
Stage II	57 (10.8)	22 (8.3)	35 (13.2)	
Stage III	123 (23.2)	67 (25.4)	56 (21.1)	
Stage IV	82 (15.5)	48 (18.2)	34 (12.8)	
Unknown	3 (0.6)	1 (0.4)	2 (0.8)	
T (%)				0.001
T1	21 (4.0)	5 (1.9)	16 (6.0)	
T1a	140 (26.4)	65 (24.6)	75 (28.2)	
T1b	110 (20.8)	61 (23.1)	49 (18.4)	
T2	55 (10.4)	23 (8.7)	32 (12.0)	
T2a	10 (1.9)	2 (0.8)	8 (3.0)	
T2b	4 (0.8)	1 (0.4)	3 (1.1)	
T3	5 (0.9)	1 (0.4)	4 (1.5)	
T3a	120 (22.6)	71 (26.9)	49 (18.4)	
T3b	52 (9.8)	23 (8.7)	29 (10.9)	
T3c	2 (0.4)	2 (0.8)	0 (0.0)	
T4	11 (2.1)	10 (3.8)	1 (0.4)	
M (%)				0.386
M0	420 (79.2)	208 (78.8)	212 (79.7)	
M1	78 (14.7)	43 (16.3)	35 (13.2)	
MX	30 (5.7)	13 (4.9)	17 (6.4)	
Unknown	2 (0.4)	0 (0.0)	2 (0.8)	
N (%)				0.566
N0	239 (45.1)	113 (42.8)	126 (47.4)	
N1	16 (3.0)	8 (3.0)	8 (3.0)	
NX	275 (51.9)	143 (54.2)	132 (49.6)	
Risk score (median [IQR])	1.15 [0.45, 2.96]	1.16 [0.49, 3.18]	1.13 [0.42, 2.65]	0.15
Risk = high/low (%)	268/262 (50.6/49.4)	135/129 (51.1/48.9)	133/133 (50.0/50.0)	0.861

**Table 2:** Detailed coefficient of the EMT-related lncRNA signature.

ID	Coef	HR	HR.95L	HR.95H	*p* value
AC084876.1	0.560669738	1.751845385	1.202464446	2.552227024	0.003497137
AL596442.2	0.265499542	1.304082258	1.098793514	1.547725312	0.002381524
IL10RB-DT	0.583163899	1.791698225	1.007267718	3.187020167	0.047188119
LINC02154	0.855956753	2.353625143	1.357881063	4.079555612	0.002287736
PSORS1C3	-0.019675941	0.980516367	0.961161745	1.000260727	0.05307191
AC002070.1	-0.466661865	0.627092097	0.474439967	0.828860393	0.001042602
LINC02027	-0.20581021	0.813987548	0.667569314	0.992519749	0.041933979
SNHG28	2.70367541	14.93452147	3.989981623	55.89998964	5.95E-05
SUCLG2-AS1	-1.005928437	0.365704943	0.103539166	1.291686137	0.118188738
AC010333.2	-2.121446142	0.119858171	0.024333434	0.590380358	0.009113773
AC068338.3	-1.209149347	0.29845105	0.153298566	0.581042805	0.000374793
AC245128.3	0.631633793	1.880680714	1.261162354	2.804523886	0.001948007

## Data Availability

The source data of this study has been uploaded to the Jianguo Yun in following website link: https://www.jianguoyun.com/p/DZwbosUQh5LQCRiR3f0D.

## Abbreviations

EMT: Epithelial-mesenchymal transition
KIRC: Kidney renal clear cell carcinoma
TKIs: Tyrosine kinase inhibitors
NGF: Nerve growth factor
VEGF: Vascular endothelial growth factor
ICIS: Immune checkpoint inhibitors
EAU: European Association of Urology
OS: Overall survival
CSS: Cancer-specific survival
MET: Mesenchymal-epithelial transition
CSC: Cancer stem cells
GC: Glucocorticoids
lncRNA: Long noncoding RNA.
